# Calibrated Link Budget of a Silicon Photonics WDM Transceiver with SOA and Semiconductor Mode-Locked Laser

**DOI:** 10.1038/s41598-017-12023-0

**Published:** 2017-09-20

**Authors:** Alvaro Moscoso-Mártir, Juliana Müller, Elmira Islamova, Florian Merget, Jeremy Witzens

**Affiliations:** 1 Institute of Integrated Photonics (IPH) of RWTH Aachen University, Sommerfeldstr. 24, D-52074 Aachen, Germany; 2 E. Islamova is now with Innolume GmbH, Konrad-Adenauer-Allee 11, 44263 Dortmund, Germany

## Abstract

Based on the single channel characterization of a Silicon Photonics (SiP) transceiver with Semiconductor Optical Amplifier (SOA) and semiconductor Mode-Locked Laser (MLL), we evaluate the optical power budget of a corresponding Wavelength Division Multiplexed (WDM) link in which penalties associated to multi-channel operation and the management of polarization diversity are introduced. In particular, channel cross-talk as well as Cross Gain Modulation (XGM) and Four Wave Mixing (FWM) inside the SOA are taken into account. Based on these link budget models, the technology is expected to support up to 12 multiplexed channels without channel pre-emphasis or equalization. Forward Error Correction (FEC) does not appear to be required at 14 Gbps if the SOA is maintained at 25 °C and MLL-to-SiP as well as SiP-to-SOA interface losses can be maintained below 3 dB. In semi-cooled operation with an SOA temperature below 55 °C, multi-channel operation is expected to be compatible with standard 802.3bj Reed-Solomon FEC at 14 Gbps provided interface losses are maintained below 4.5 dB. With these interface losses and some improvements to the Transmitter (Tx) and Receiver (Rx) electronics, 25 Gbps multi-channel operation is expected to be compatible with 7% overhead hard decision FEC.

## Introduction

Dense (D)WDM is being considered as one of the approaches to further scale the data throughput of SiP transceivers. Major difficulties then reside in generating a sufficient number of optical carriers in a compact form factor as well as in spectrally aligning optical carriers with other optical devices such as filters and multiplexers, the large thermo-optic coefficient of Silicon (Si) making some form of thermal stabilization a prerequisite for the latter. A promising approach for generating a large number of optical carriers on a DWDM grid consists in the heterogeneous integration of lasers into SiP chips during Front-End-Of-Line (FEOL) fabrication^1,2^, even though this also raises further issues in regards to heatsinking of the light sources. Here, we investigate another approach relying on a single semiconductor MLL^3^ utilized as a multi-carrier light source, in which mode-locking is used to suppress mode partitioning noise. Advantageously, this approach allows fabrication of the SiP chips without requiring chip/wafer bonding or introduction of III-V materials into the FEOL, as well as the assembly of a known good SiP die with a known good laser downstream in the manufacturing process. However, it also comes with its own set of challenges related to the stabilization of MLL operation, relatively low comb line power levels and relatively high Relative Intensity Noise (RIN) levels recorded once individual comb lines have been separated. In a conventional WDM system relying on an array of Distributed Feedback Lasers (DFB), each laser can source several tens of mW with a typical RIN on the order of −135 dBc/Hz. On the other hand, the single section semiconductor MLLs utilized here source on the order of 1 mW per comb line and isolated comb lines feature a substantially higher RIN, on the order of −120 dBc/Hz for frequencies below 4 GHz^4^. As will be shown in the following, this puts considerable strain on the link budget, so that downstream amplification after modulation becomes necessary^4^. Here, amplification by means of an SOA will be evaluated.

A number of recently demonstrated compact form factor SiP WDM systems have been designed for channel counts that are multiples of four^5,6^ in line with recent Multi-Source Agreements (MSAs) for Electro-Optic (E/O) datacenter transceivers, such as Quad Small Form-Factor Pluggable (QSFP) or C Form-Factor Pluggable (CFP) four-channel modules, eight-channel CFP2 modules or 12-channel CXP modules. Serial data rates up to 28 Gbps support Infiniband Extended Data Rate (EDR), 4-lane 100 G Ethernet and 32 G Fibre Channel. Here, we are aiming at 8- and 12-channel solutions relying on a single MLL and a single SOA. With typical power consumptions of respectively 360 mW and 780 mW, the MLL and SOA consume a total of 1.1 W of electrical power, or an equivalent of 95 mW per channel in a 12-channel configuration. Assuming a wall plug efficiency of 15%, a standalone DFB would have generated 14 mW of optical power, which is on the order of what is required to sustain a non-amplified Silicon Photonics optical link^7,8^. Furthermore, we focus on the use of Resonant Ring Modulators (RRMs) in the SiP Tx subsystem. Since they advantageously combine modulation functionality with frequency selective operation, they can be ideally paired with comb sources without additional multiplexers being required in the Tx. As a drawback, RRMs require thermal stabilization to maintain their spectral alignment with their corresponding carriers. While this is a very significant drawback in a parallel single mode (PSM) transceiver solution, since maintaining the operating point of an RRM is generally considered an intractable problem in a thermally free running solution, in a DWDM transceiver as pursued here some amount of global thermal stabilization of the module via a Peltier element is unavoidable so that the additional power overhead associated to RRM stabilization is much reduced. Other modulator types such as Mach-Zehnder Modulators (MZM) are not analyzed in detail here, however the derived link budget models can be straightforwardly adapted.

In this paper, we model a comprehensive link budget for an SiP DWDM link combining a semiconductor single section MLL as a multi-carrier light source with RRMs followed by a single polarization quantum well SOA on the Tx side, with the particular aim of providing compact and power efficient WDM solutions for short distance Datacom interconnects. In ref.^9^ we have experimentally investigated such a link implemented with SiP devices operated in single channel configuration. As a key result, we experimentally showed that error free operation, defined here as a Bit Error Ratio (BER) below 1e-12, could be reached at 14 Gbps in the targeted architecture without FEC, while 25 Gbps required some amount of FEC with 5 dB coding gain (defined as the improvement to the square of the signal quality (Q-)factor expressed in dBQ) in order to maintain error free operation with a typical 0 dBm comb line power. The key contribution of this paper is the investigation of penalties associated to multi-channel operation, in particular channel cross-talk and penalties related to partial SOA saturation, the resulting data distortion due to pattern dependent gain (XGM), as well as other nonlinearities (FWM).

The paper is organized in four sections: In the first section, the investigated Tx and Rx architectures are described. The second section focuses on the SOA and on the models used to account for SOA saturation, in particular XGM. Further modeling assumptions and a high-level description of the comprehensive link modeling results are given in section 3. Finally, the fourth section provides some general design guidelines for comb source based and SOA amplified WDM links, as well as an outlook on further work. Details of the other components assumed to be utilized (MLL, RRM, Ge photodiode) can be found in ref.^9^ and its Supplementary Materials. Further details on models accounting for FWM and optical cross-talk can be found in the Supplementary Materials of this paper.

One of the main objectives is to evaluate acceptable optical insertion losses (ILs) at laser-to-SiP-chip, SiP-chip-to-SOA and SOA-to-SiP-chip interfaces, as well as to evaluate the channel count scalability of the technology. It should be noted that impairments due to fiber dispersion are not analyzed here, as the focus of this work lies on short distance Datacom links. However, experimental characterization of the RRMs assumed to be utilized in this paper showed no additional Intersymbol Interference (ISI) penalty for increased fiber lengths up to 10 km when driven with the 2 V_pp_ signal assumed here (at 6 V_pp_ drive signals, a modest 0.5 dB closure of the vertical eye opening was recorded at 10 km fiber length). A comprehensive study of RRM induced chirp and related limitations on long distance transmission can be found in ref.^10^.

## Architecture Overview

**Figure 1:**
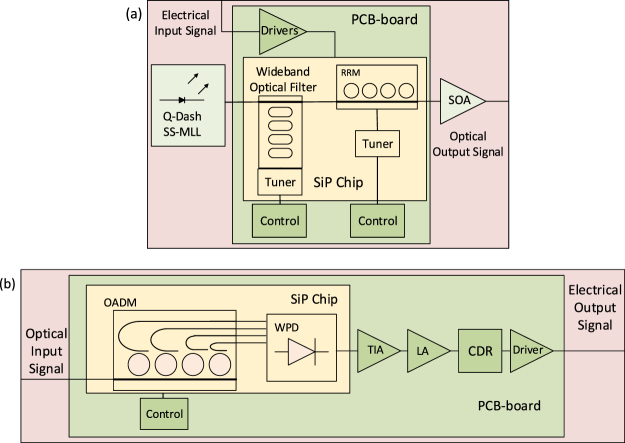
Intended integrated Tx (top) and Rx (bottom) architectures: SiP chip with Printed Circuit Board (PCB) including drivers and control electronics, as well as hybridly integrated MLL and SOA. Rx comprising Optical Add-Drop Multiplexers (OADM), Waveguide Photodiodes (WPD), Transimpedance Amplifiers (TIA), Limiting Amplifiers (LA), and Clock-Data Recovery (CDR).

The targeted Tx and Rx architectures are shown in Fig. 1. The Tx comprises an MLL with a 100 GHz Free Spectral Range (FSR) coupled to an SiP chip. Since a downstream SOA is meant to be operated in the linear regime or at the onset of saturation, after entering the chip unused comb lines are removed by a wideband filter so as to ensure the available SOA output power can be optimally allocated to the modulated lines. This wideband filtering occurs before modulation since this reduces the requirements on the filter’s spectral alignment and passband edge steepness. The remaining carriers are then modulated by an RRM array optimized for a drive voltage of 2 V_pp_ (0 V to 2 V reverse bias). The electrical signal is generated by a driver from Mellanox Technologies with integrated Clock Data Recovery (CDR) and signal reshaping. Optional pre-emphasis of the optical channel as implemented in the driver is not used as the bandwidth of the E/O devices is sufficient to sustain 25 Gbps signaling as is^9^. Subsequently, all optical channels are jointly amplified by an SOA prior to being sent to the Rx. Amplification after modulation allows for compensation of the high attenuation induced by the RRMs operated close to resonance (low optical carrier detuning, referring to the frequency detuning of the optical carrier relative to the resonance) with relatively low electrical signal levels. Amplification prior to modulation on the other hand would not help much due to the limited output power of the SOA. Furthermore, boosting the optical power levels prior to sending the light through the RRMs could lead to difficulties related to bistabilities and self-pulsation inside the rings that emerge as the optical power levels increase^11^ which would be further exacerbated here by operating the RRMs close to resonance (as required for a high signal extinction).

The targeted Rx architecture consists in an optical demultiplexer implemented in the form of cascaded ring based Optical Add-Drop Multiplexers (OADMs) each routing a channel to an individual drop waveguide connected to a separate photodiode. In this architecture, the OADMs also filter out Amplified Spontaneous Emission (ASE) and contribute to ASE-ASE beat noise reduction.

The link budget is calibrated based on the experimental single channel characterization of a similar link reported in ref.^9^: SiP Tx chips containing the targeted RRM design and SiP Rx chips containing either a Germanium (Ge) Waveguide Photodetector (WPD) or a flip-chip photodiode (FC-PD) array have been used in conjunction with 25 Gbps chip-scale driver and receiver electronics and connected via polarization maintaining fiber for single-channel experiments. Additional penalties arising from full photonic chip-scale integration (in particular, integration of the OADMs), handling of polarization diversity and WDM operation – in particular those associated to optical cross-talk, SOA saturation and other nonlinear effects inside the SOA – are introduced into a comprehensive link budget via modeling. The assumed SOA characteristics are based on the detailed characterization of nonlinear effects in the commercial quantum well based SOA with which the system experiments have been performed (Thorlabs S9FC1004P).

### Modeling of SOA Nonlinearities

Since the intent of this work is to support the maximum number of channels with the minimum number of active devices, the SOA will be typically operated close to or at the onset of saturation, and will thus leave the linear regime when implementing the full WDM link. Unlike Erbium Doped Fiber Amplifiers (EDFAs), SOAs have a much shorter carrier lifetime, below a nanosecond, so that the gain relaxation is sufficiently fast to track the data stream when the SOA enters saturation. This leads to a well-known high pass behavior with a higher output modulation amplitude at signal frequencies above a transition frequency in the GHz range. In addition, FWM leads to additional inter-channel cross-talk and to further penalties not seen in the single-channel experiments described in ref.^9^. While these effects have been well documented in the literature^12,13^, we summarize here the models and assumptions entering the link budget calculations whose results are reported in the next section.

#### SOA Saturation and Cross-Gain Modulation

**Figure 2:**
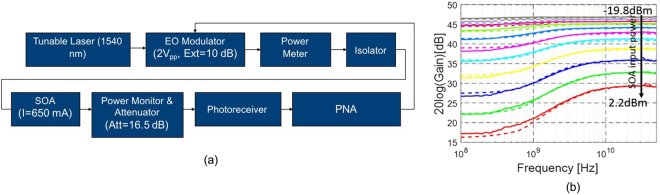
(**a**) SOA characterization setup. (**b**) Measured (continuous lines) and modeled (dashed lines) S_21_ of the SOA operated with a single channel for different average power levels reported at the input of the packaged SOA (8 dB signal extinction).

**Figure 3:**
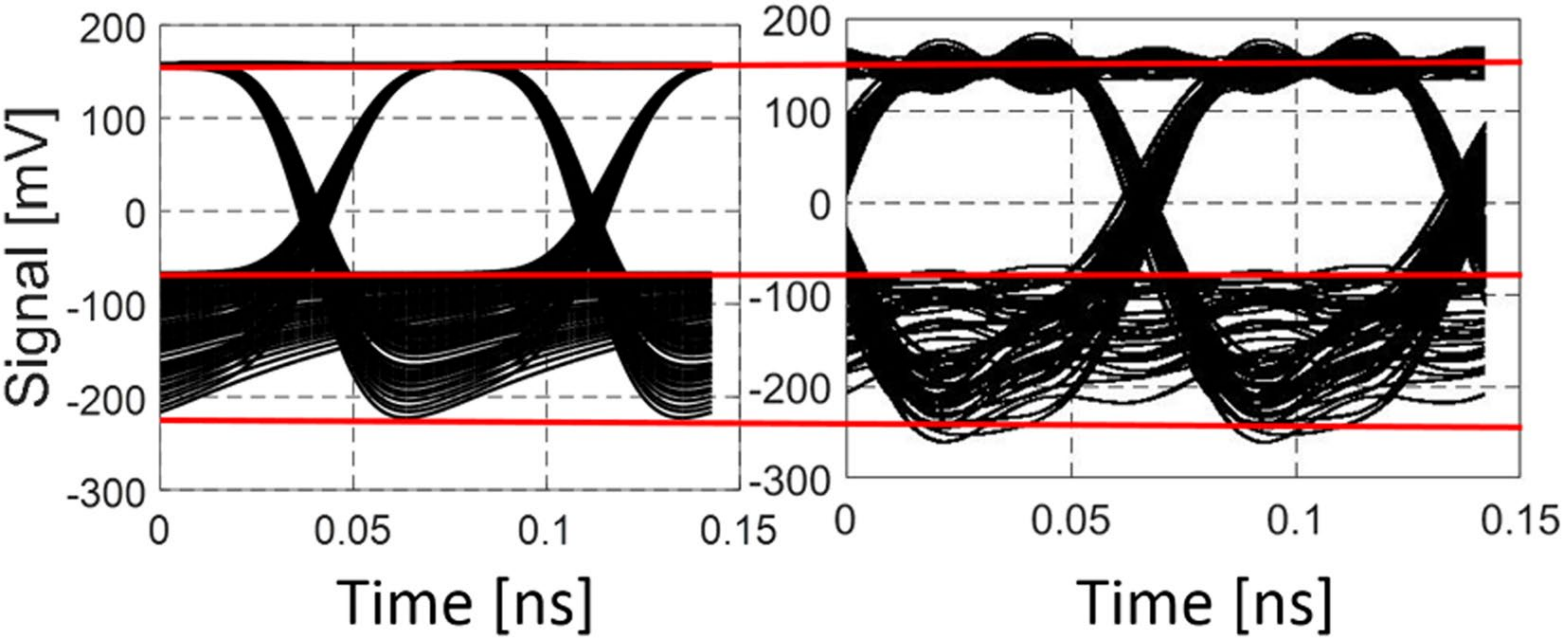
(**a**) Measured and (**b**) calculated 14 Gbps eye diagrams after single channel amplification by the SOA for an average power of −7 dBm and a signal extinction of 17 dB, both specified at the input of the packaged SOA. This corresponds to a typical configuration assuming 12 fully correlated channels under the assumptions of section III.

 In order to quantify the saturation effects occurring in the utilized SOA, we measure its S_21_ by amplifying a signal (8 dB extinction) generated by a tunable laser source and a commercial MZM. Before entering the commercial photoreceiver, the signal is attenuated by 16.5 dB to avoid saturation of the Rx (Fig. 2(a)). The average power entering the packaged SOA is varied from −19.8 to 2.2 dBm to record different levels of saturation (here and throughout the paper, the durations of ones and zeros are assumed to be equal, so that the average power is simply given by *P*_*AV*_ = (*P*_1_ + *P*_0_)/2 with *P*_1_ the 1-bit power and *P*_0_ the 0-bit power). The data is normalized by measuring the S_21_ of the same link after removing the SOA (with the optical attenuation adjusted to ensure the same average power enters the photoreceiver, since we found the cutoff frequency of the commercial Rx utilized in this experiment to be input power dependent). The measurements are fitted with solutions to the following well-known differential equation by adjusting the gain relaxation time constant ${\tau }_{c}$

1 $$\frac{dG}{dt}=-\frac{G-{G}_{0}}{{\tau }_{c}}-\frac{G{P}_{SOA,in}}{{P}_{sat,in}{\tau }_{c}}$$

where *G*_0_ is the small signal gain at low average input powers and is 24 dB for the packaged SOA (30 dB for the bare die accounting for fiber coupling losses and an interposed isolator at both the input and output ports). *P*_*sat,in*_, the saturation input power of the SOA at the 3 dB gain compression point, is −6 dBm for the packaged SOA (−9 dBm for the bare die, again accounting for fiber coupling losses and an interposed isolator). A *τ*_*c*_ of 0.17 ns was found to result in a good agreement with the measured S_21_ curves displayed in Fig. 2(b). Subsequently, we measured eye diagrams at 14 Gbps with the same experimental setup to confirm the validity of the modeling in the large signal regime. The signal extinction was changed to 17 dB and the average power set to −7 dBm. This configuration corresponds to the typical input power and extinction of 12 fully correlated channels under the assumptions of Table 1 in the following link modeling section (as discussed in the following, all channels synchronously transmitting an identical data stream constitutes a worst-case assumption in regards to XGM). The eye diagram is reconstructed with the help of Eq. (1) and converted to the electrical domain by taking the power dependent (negative) conversion gain of the photoreceiver into account. Figure 3 shows a comparison of the measured (a) and the modeled (b) eye diagrams. Generally, there appears to be a good agreement between the actual and calculated signal levels. Moreover, SOA saturation is seen to result in some amount of signal distortion in this configuration.

**Table 1:** List of device and system characteristics (modeled full WDM link). *P*_*sat,out*_ refers to the SOA output power at the 3 dB gain compression point.

Quantity	Value	Comment
MLL to Tx IL	*IL*_*A*_	Scanned
On-Chip Tx Filter IL	1 dB	Simulated
Tx Filter Extinction	17 dB, 15 dB	Simulated for 8 & 12 channels
RRM Ext.	17 dB	Exp. data from ref.^9^, Fig. SM4(b)
RRM MP	10.9 dB	Exp. data from ref.^9^, Fig. SM4(b)
RRM Cutoff Freq.	18.5 GHz	Based on device models
IL off-channel RRMs	0.5 dB	Calculated based on Q-factors
Driver Rise/Fall Time	16 ps	From driver specifications
Tx to SOA IL	*IL*_*A*_	Scanned
SOA Gain @ 25 °C	27 dB	Bare die, below specification
SOA Gain @ 45 °C	24 dB	Bare die, 30–6 dB
SOA Gain @ 55 °C	21 dB	Bare die, 30–9 dB
SOA NF @ 25 °C	6 dBe	Bare die, as specified
SOA NF @ 45 °C	7.5 dBe	Bare die, 6 + 1.5 dBe
SOA NF @ 55 °C	8.25 dBe	Bare die, 6 + 2.25 dBe
SOA *P*_*sat,out*_ @ 25 °C	18 dBm	Bare die, as specified
SOA *P*_*sat,out*_ @ 45 °C	17 dBm	Bare die, 18–1 dBm
SOA *P*_*sat,out*_ @ 55 °C	16.5 dBm	Bare die, 18–1.5 dBm
SOA NF Penalty @ *P*_*sat,in*_ −2 dB	2 dBe	Based on ref.^14^
SOA NF Penalty @ *P*_*sat,in*_ + 0 dB	3 dBe	Based on ref.^14^
SOA NF Penalty @ *P*_*sat,in*_ + 3 dB	4 dBe	Based on ref.^14^
SOA to Tx IL	*IL*_*A*_	Scanned
FWM Penalty	0.4 dB	Estimated from measured FWM
Tx to Fiber IL	3 dB	Measured (perm. attached GC)
Fiber to Rx IL	5 dB	Literature review
On-Chip OADM IL	0.85 dB	Measured^4^
OADM FWHM	40 GHz	Targeted (${\rm{\Delta }}{f}_{OF}$ = 20 GHz)
Ge WPD Int. Resp.	0.7 A/W	On-chip resp., as measured^9^
TIA Inp. Ref. Noise	2 μA	From meas. Rx noise floor^9^
TIA Cutoff Frequency	21 GHz	3^rd^ order Butterworth, from sims.
Calc. ISI Penalty	0.76, 1.7 dB	Calc. @ 14 & 25 Gbps
Excess Rx Penalty	0, 2.65 dB	Measured^9^
Inter-Channel X-talk Eye Opening Penalty	0.5 dB	Calc. for 25 Gbps based on spectral overlap with OADM bandwidth

#### Quantification of Resulting Link Penalties

Determining the impact of SOA saturation on the link is not entirely straightforward, since the gain saturation seen by one channel depends on all the channels sent through the SOA. We are making a few simplifications in that regards that ought to be spelled out: A first approximation consists in the simplified SOA model given by Eq. (1) attributing the gain dynamics to a single time constant. Furthermore, it makes the assumption that the gain coefficients of all the channels follow the same time dependency derived from the aggregate power entering the SOA. Physically, this is an adequate assumption as long as gain dynamics are primarily determined by Carrier Density Pulsation (CDP). It does, however, neglect channel specific gain variations resulting from Spectral Hole Burning (SHB) and Carrier Heating (CH), which is an appropriate assumption here since, at the time scales of the data stream, gain saturation and XGM are dominated by CDP.

Another important assumption resides in the degree of correlation assumed amongst the channels: Indeed, the worst case for XGM corresponds to all the channels synchronously transmitting an identical data stream (which would then result in the largest swing of the dynamic gain). This is, however, a very rare occurrence. A sophisticated analysis would take into account the binomial distribution describing all possible combinations over the characteristic SOA gain relaxation time constant. This analysis would however presuppose knowledge of the degree of independence between the channels. Here, we analyze two extremes: The worst-case situation corresponding to *N* perfectly correlated channels, as well as the best-case situation corresponding to the *N* channels being exactly balanced in terms of the number of transported zeroes and ones. A maximum disparity between the number of zeroes and ones synchronously transmitted amongst the channels could be guaranteed with some encoding overhead, for example by transporting 8 logical channels over 10 optical channels after applying 8b/10b encoding. Reduced power comb lines outside of the center region of the MLL spectrum with insufficient power to reliably transport a data stream could be leveraged to balance out the average power without reducing the number of available channels.

A very important benefit of keeping the overall power entering the SOA constant, that is even more important than preventing the aforementioned reduction of the vertical eye opening, consists in maintaining a high signal extinction even in presence of partial SOA saturation. This is achieved in the balanced case since the gain remains constant over time. Indeed, while the reduction of the vertical eye opening is the relevant metric to assess the reduction of signal quality as limited by additive Rx noise, this additional eye closure does not result in a one-to-one reduction of the signal quality in a RIN and ASE limited link: If we make the simplifying assumption that the Noise Figure (NF) of the SOA does not change significantly at the onset of saturation, the quality of a signal with infinite extinction would remain the same, as RIN and ASE are amplified by the same reduced gain as the 1-level signal. A reduced extinction on the other hand directly results in increased ASE noise and RIN, as compared to the Optical Modulation Amplitude (OMA), and is thus the relevant metric to quantify the effect of SOA saturation in a RIN and ASE limited link. Due to the relatively low launch power (for an SiP based solution with its high interface losses) and the high RIN of isolated comb lines^9^, RIN and ASE indeed play a dominant role in the investigated link. Consequently, the reduction of extinction in the imbalanced (worst) case turned out to be a critical factor in the link budget analysis reported in the next section. Note that in practice the NF also worsens as the SOA enters saturation and becomes signal level dependent due to a reduction of the population inversion, see for example^14^, an effect that is also taken into account in the full link model of section III.

The two scenarios are modeled as follows: In case of balanced channels, the constant gain of the SOA is simply given by

2 $$G=\frac{{G}_{0}}{1+\frac{N({P}_{SOA,0}+{P}_{SOA,1})}{2{P}_{sat,in}}}$$

where ${P}_{SOA,0/1}$ are the power levels of the 0- and 1-states at the input of the SOA. Assuming the NF to remain unchanged, RIN, ASE and signal are all reduced by the same multiplier at the entrance of the Rx. Since the Rx input referred noise remains unchanged, it is effectively increased by a factor $1+N({P}_{SOA,0}+{P}_{SOA,1})/2{P}_{sat,in}$ relative to the signal strength.

In the worst-case situation, we assume the gain to be largely determined by the other synchronized *N*−1 channels and to be largely uncorrelated to the data stream of the investigated channel. The gain then drifts between two extremes given by

3 $${G}_{min}=\frac{{G}_{0}}{1+\frac{N{P}_{SOA,1}}{{P}_{sat,in}}},{G}_{max}=\frac{{G}_{0}}{1+\frac{N{P}_{SOA,0}}{{P}_{sat,in}}}$$

Since the gain saturation is now largely independent of the bit sequence of the investigated channel, associated penalties can be simply stacked with ISI penalties associated to bandwidth limitations in the single-channel model, without having to take data sequence dependent correlations into account. The SOA saturation induced eye opening penalty (in dB) is then given by

4 $$-10lo{g}_{10}(\frac{{G}_{min}{P}_{SOA,1}-{G}_{max}{P}_{SOA,0}}{{G}_{0}({P}_{SOA,1}-{P}_{SOA,0})})$$

increasing, as previously, the Rx noise in relative terms (by a larger amount than in the balanced case). The reduced extinction (also in dB) is further given by

5 $$10lo{g}_{10}(\frac{{G}_{min}{P}_{SOA,1}}{{G}_{max}{P}_{SOA,0}})$$

and now enters the link budget in the evaluation of the increased 0-level (and relative 1-level) RIN and ASE noise.

**Figure 4:**
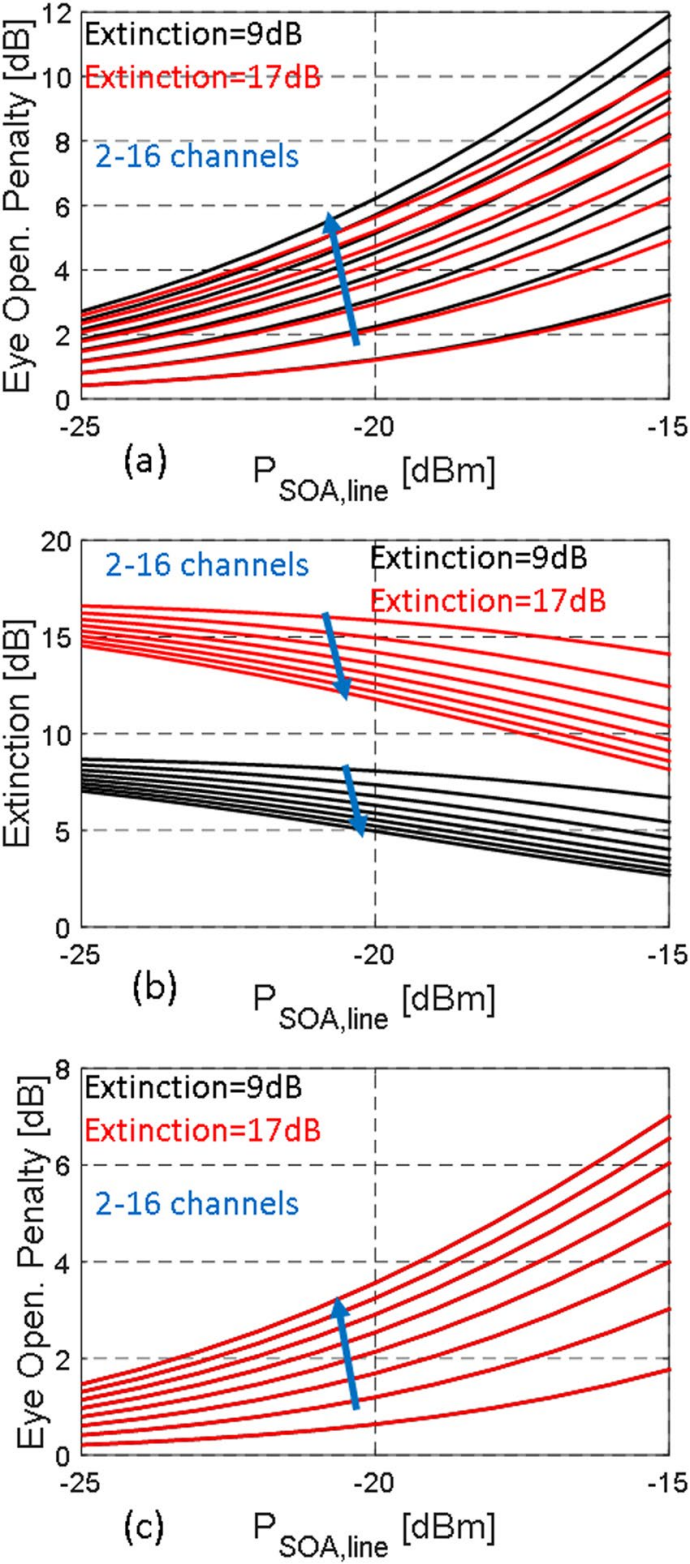
Eye opening penalties due to partial SOA saturation for the worst case (**a**) and balanced (**c**) scenarios as a function of the number of channels and of the average input power per channel (specified here *after* coupling to the SOA chip). (**b**) Shows the reduction of the extinction due to partial SOA saturation for the worst-case scenario (for the balanced case the extinction is not modified, so that it is not drawn here). The black and red curves respectively correspond to an initial signal extinction of 9 and 17 dB prior to entering the SOA.

Figure 4 shows the vertical eye opening penalty and the penalized extinction computed for both scenarios as a function of channel count (2 to 16 channels in steps of 2, assuming all other comb lines to be fully filtered out prior to reaching the SOA) and of the average power per line after coupling to the SOA chip (i.e., after factoring in incoupling losses). It forms the basis for the inclusion of SOA saturation based penalties in the next section. We also plot the data for two assumptions of pre-SOA extinction, 9 and 17 dB. As discussed in the next section, in the assessment of the multi-channel operation we will assume the optical carrier detuning to be even further reduced compared to the experiments reported in ref.^9^, increasing the extinction to 17 dB and further reducing average power after the RRMs, at the cost of some increase of the Modulation Penalty (MP)/reduction of the OMA. When reading the graph, one should keep in mind that the typical average power per line at the input of the SOA (after coupling to the SOA chip) will then be below −21 dBm in this scenario, assuming 0 dBm sourced per MLL comb line, MLL and SOA interface losses (*IL*_*A*_) of at least 3.5 dB each, and an attenuation of the 1-level power by 11 dB by the RRM (consistent with the required optical carrier detuning to reach 17 dB extinction). Figure 4 clearly shows a reduction of the extinction and eye opening as the number of channels is increased. This effect is more pronounced when all the channels are synchronously transmitting (worst case), because the large swing in the total SOA input power affects its gain when operated in saturation. In the balanced case, the degradation of the eye opening only depends on average input power and is thus independent on the initial extinction level, as can be seen in Fig. 4(c) (the red and black curves overlay).

Channel penalties associated to FWM inside the SOA are also included in the link model described in the next section. We estimate these to be rather small (0.4 dB) in the regime of operation assumed for the WDM link, so they are not further discussed here. For a detailed description of the FWM analysis, please refer to the Supplementary Materials.

As a final note, one should mention that reduction of RIN by means of a saturated SOA, as has been used in the literature in single-channel configurations^15^, would not be applicable here. This is not due to the multi-channel operation per se, but rather to the way the RIN of individual comb lines correlate with each other in a typical semiconductor comb laser: The RIN of the entire comb is orders of magnitude smaller than the RIN of isolated channels^4^, so that the SOA gain cannot be expected to correlate to any single channel RIN once a large number of channels are transmitted jointly through the SOA.

### Multi-Channel Transceiver Link Budget

A number of modifications are introduced here relative to the calibrated full link model used to reproduce the experiments in ref.^9^: RIN, as measured on the individual MLL comb lines and reported in ref.^9^, is reintroduced into the link model. The filter interposed between the SOA and the photodiode is assumed to be implemented in the form of a single ring based OADM integrated inside the Rx SiP chip with a Lorentzian shaped transfer function. Excess losses associated to managing polarization diversity with a Polarization Splitting Grating Coupler (PSGC) at the input to the Rx are taken into account. Penalties associated to SOA saturation, XGM and FWM as described in the previous section are further introduced into the model. An integrated wideband filter is assumed to be interposed between the MLL and the RRMs on the Tx chip in order to select optical carriers. The performance of this filter has been assessed based on device simulations of a Coupled Resonator Optical Waveguide (CROW) based design that will be published separately. Note that due to the low 1-level power transmitted by the RRM operated with 2 V_pp_ at a low optical carrier detuning (the 1-bit level is extinguished on the order of −7.5 to −11 dB by the RRM for typical bias points), the extinction of the unused comb lines has to be quite high for them not to unnecessarily contribute to SOA saturation. Finally, the MLL and SOA are assumed to be hybridly integrated into the Tx, with the SOA coupled back to the Tx SiP chip prior to light being grating coupled to the output fiber, so that it has two optical interfaces with the SiP chip. Since the MLL-to-SiP-chip, SiP-chip-to-SOA and SOA-to-SiP-chip interface losses are not yet known (the hybrid integration process is still under development), these losses are parameterized in the following and the maximum acceptable interface losses determined.

#### Penalties Associated to Component Bandwidths, ISI and Optical Cross-Talk

The analysis of the effect of the OADM optical filter bandwidth on the performance of the link can be found in the Supplementary Materials. A tradeoff has to be struck between ISI, a minimization of which requires a larger filter bandwidth, and inter-channel cross-talk to adjacent channels, a minimization of which requires a smaller filter bandwidth. A smaller filter bandwidth is also beneficial in reducing the ASE-ASE beat noise. The effect on ASE-signal beat noise is less as the latter is already filtered by the Opto-Electronic (O/E) bandwidth of the Rx, but nonetheless here too the OADM filter bandwidth still plays a role in the overall Noise Equivalent Bandwidth (NEB). While higher order filters with steeper roll-offs would facilitate accommodating both low optical cross-talk and low ISI^16^, we are still basing the analysis on single ring Lorentzian filters here in view of maintaining a manageable complexity for the complete solution. As described in the Supplementary Materials, an OADM bandwidth of 40 GHz, corresponding to the Full Width at Half Maximum (FWHM) of the ring, is close to optimum in this link architecture for 25 Gbps serial signaling.

In this section, the Tx is modeled as a chip-scale driver with a finite rise and fall time (16 ps for 20% to 80% transitions as per specifications) followed by a single pole filter modeling the transfer function of the RRM. The latter is modeled based on measured characteristics (RRM2 in ref.^9^), but assuming a lower optical carrier detuning (4.4 GHz) than in the system experiments reported in ref.^9^ in order to obtain a larger extinction (17 dB) at the cost of a reduced cutoff frequency (18.5 GHz) and increased MP (10.9 dB), defined as the OMA reduction caused by the finite drive voltage. Simulations of the linear frontend of the TIALA can be fitted well by a third order Butterworth filter with a 21 GHz cutoff frequency. The photodiode itself is assumed not to further limit the Rx bandwidth, as it is assumed to be the low capacitance Ge WPD described in ref.^9^ with an intrinsic bandwidth above 30 GHz.

#### Penalties Associated to Polarization Management

For managing polarization diversity at the Rx, we are considering the use of a PSGC. While PSGCs have higher IL than single polarization Grating Couplers (GCs), on the order of 6 dB^17^ when fabricated in a standard 220 nm device layer thickness Silicon-On-Insulator (SOI) wafer, a number of strategies have been successfully implemented to reduce these ILs. Higher insertion efficiency can be obtained by modifying the thickness of the Si device layer either by modification of the SOI wafers^18^ or by depositing a poly-Si layer on top of a standard 220 nm device layer^19^. Further improvements have also been obtained by increasing the directionality of the GCs with help of dual SOI stacks^18^ or the deposition of a metal back-reflector^20^. PSGC IL as low as 2.8 dB (without dual SOI stack) and 2.2 dB (with dual SOI stack), both in 310 nm device layers, have been experimentally demonstrated. Here, we found that at room temperature the link budget is primarily limited by ASE and RIN due to the high gain of the SOA, so that we can assume a conservative value for the PSGC IL without excessively penalizing the link. We are assuming 5 dB IL in the following, corresponding to a modest improvement relative to the 6 dB reported in^17^, particularly once permanent attachment of the fiber with an index matched epoxy is taken into consideration that already results in a ~0.5 dB improvement. Lower PSGC losses would of course allow dialing down the SOA gain (by shortening the device) and thus be conducive to increasing the channel count, as a same amount of SOA output saturation power (in a given SOA technology) could be allocated amongst a larger number of channels. While a PSGC adds excess IL at the input of the Rx compared to a single polarization GC, we do not expect a worsening in regards to ASE noise in the dual polarization receive configuration: Since the SOA used at the Tx is quantum-well based and thus generates ASE in only one polarization, summation of both polarizations at the photodetector^21^ will not lead to an increase of the recorded ASE noise.

#### Summary of Assumptions

Table 1 summarizes the assumptions made to evaluate the link budget of the transceivers based on the proposed architecture. Since the SOA and laser attachment processes are still under development, a large uncertainty remains in regards to the IL that will be achievable (first results can be found in ref.^22^). For this reason, no single assumption is made for the attachment losses (noted as *IL*_*A*_ in the following). Rather, they are scanned together with MLL RIN and line power in order to map the acceptable parameter space. For reduction of both 0-level RIN and signal-ASE beat noise, a high signal extinction is important. RRM characteristics assume an even further reduced optical carrier detuning than in the system experiments reported in ref.^9^, but correspond to experimental data recorded from RRM2.

The “Excess Rx Penalty” corresponds to a worsening of the Rx sensitivity observed in ref.^9^ when increasing the datarate from 14 Gbps to 25 Gbps, which was attributed to a limitation of the utilized chip-scale electronics. Two further items in this table require further discussion: The temperature dependent SOA characteristics and the “Tx Filter Extinction”.

#### Interplay Between SOA Gain, SOA Saturation, and Wideband Optical Channel Selection Filter

At room temperature, the assumed SOA gain has been *reduced* from the 30 dB specified for the commercially available bare die to 27 dB, assuming a shorter SOA with lower gain but similar output saturation power. Reducing this high gain does not penalize the single-channel link budget much (at room temperature the noise budget is dominated by ASE and RIN so that Rx noise only plays a small role), while it enhances the channel count scalability given the available SOA output saturation power (18 dBm output power at the 3 dB gain compression point for the bare die). On the other hand, at increased temperatures we evaluate the available gain by taking the 30 dB specified at room temperature as a baseline and subtracting the temperature dependent gain reduction. In other words, the available commercial SOA has adequate sizing at the increased temperature operation of a semi-cooled module. Indeed, as discussed below, at 55 °C the link budget is equally limited by RIN, ASE and Rx noise given these SOA characteristics. The 6 dB gain reduction at 45 °C was measured and the 9 dB gain reduction at 55 °C extrapolated. The 1 dB reduction in output saturation power at 45 °C is also based on a measurement, while the increase in the NF is based on a typical temperature dependence reported in the literature^23^.

The “Tx Filter Extinction” is actually a system metric that combines CROW filter and MLL characteristics: By this, we refer to the amount of optical power from all the unused comb lines transmitted to the output of the filter divided by the total power of all the comb lines used as optical carriers, also at the output of the filter. This factors in the shape of the spectrum of the MLL assuming that the strongest central lines are used as carriers. The finite extinction of the unused lines by the CROW filter results in an increased level of SOA saturation relative to a perfect filter. Its evaluation incorporates the results of a sensitivity analysis based on typical fabrication variations. Importantly, at the system level these extinctions are not as high as would first appear, as the optical carriers are further attenuated by the RRMs, while the unused carriers are not. If we take into account that the average carrier power is further attenuated by 14 dB, these Tx Filter Extinctions (respectively evaluated for the independently designed 8- and 12-channel versions of the filter) drop to 3 and 1 dB. I.e., at the input of the SOA the additional unused optical power from unmodulated lines is respectively 50% and 80% of the average “useful” optical power corresponding to modulated optical carriers. Importantly, as shown in the following, this (initially unwanted) additional constant power is not as detrimental as one could first assume, as it also contributes to stabilizing the SOA input power (particularly in the worst-case assumption of fully correlated channels) and thus also reduces the deterioration of the signal extinction.

#### Wavelength Dependencies

A number of devices have finite optical passbands that limit the range of operation of the system. 12 channels with a 100 GHz spacing cover a 9.6 nm wavelength range that has to fit into these passbands. Single polarization GCs typically have a 30 nm −1 dB penalty passband, while polarization splitting GCs typically have a somewhat reduced bandwidth, on the order of 20 nm, due to polarization dependent losses. Additional insertion losses associated to these finite passbands are assumed to be included in the GC insertion losses summarized in Table 1. The utilized SOA has a 3 dB gain bandwidth of 85 nm. The gain assumed in Table 1 is actually slightly below the maximum gain and can be maintained in a 30 nm wavelength range. The assumed Ge WPD responsitivity was measured at 1550 nm^9^. At room temperature, the 15 high power comb lines of the measured MLL whose characteristics are taken as the basis for this analysis were all below 1550 nm. At 70 °C, the laser spectrum would be red shifted, but remain below 1560 nm, which would result in a ~1 dB responsivity penalty due to a reduced Ge absorption coefficient. The center wavelength of the laser could however be straightforwardly retargeted to maintain all the modulated lines below 1550 nm at the operating temperature.

#### Discussion of Results

**Figure 5:**
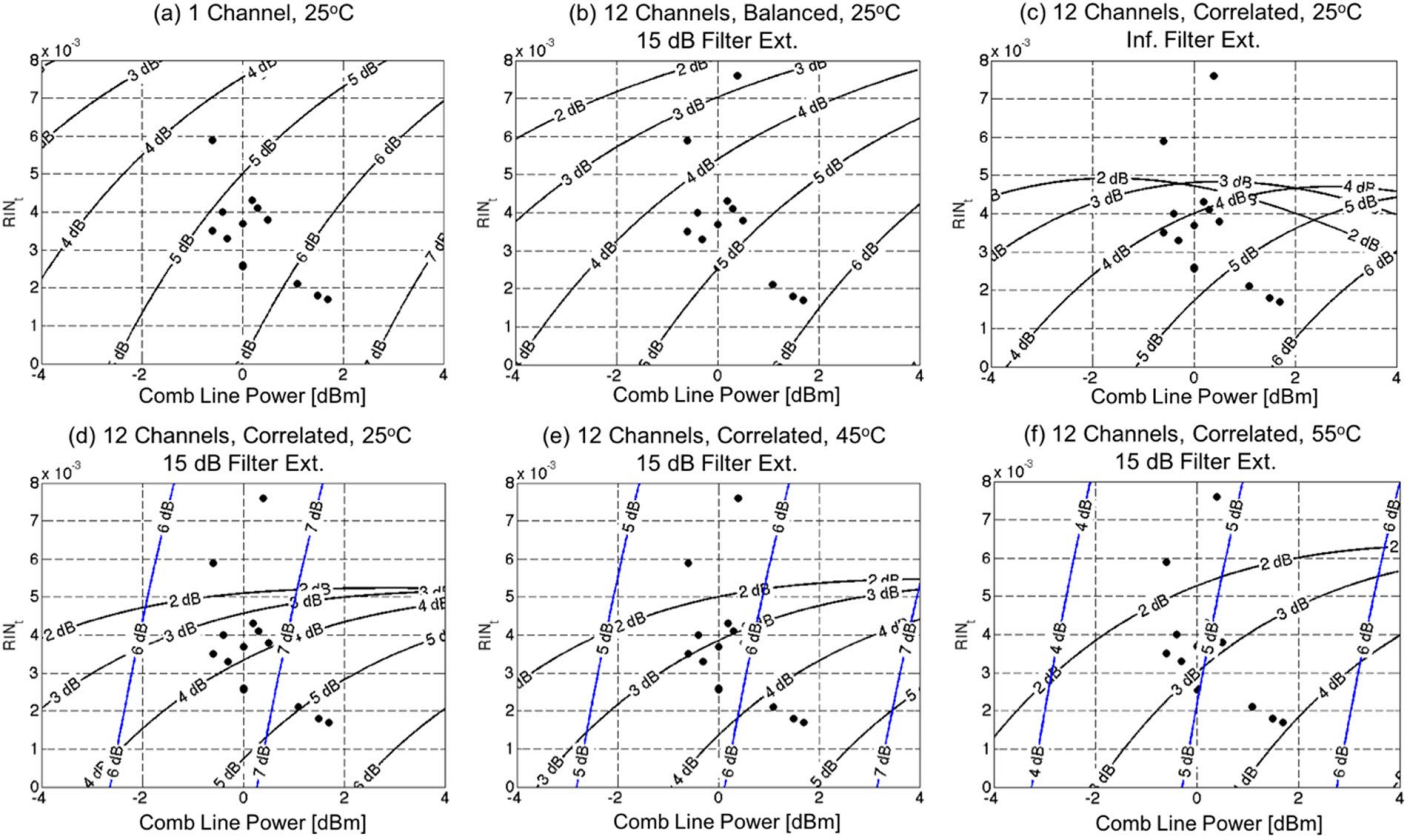
Maximum integrated comb line RIN and minimum required comb line power as a function of the MLL-to-SiP and SOA-to-SiP interface losses (*IL*_*A*_) at 14 Gbps serial data rates assuming (**a**) no SOA saturation and (**b**–**f**) SOA saturation with (**b**) 12 balanced channels and 15 dB Tx Filter extinction (**c**) 12 correlated channels and infinite Tx Filter extinction and (**d**–**f**) 12 correlated channels and 15 dB Tx Filter extinction with the SOA operating at (**a**–**d**) 25 °C, (**e**) 45 °C and (**f** ) 55 °C. The dots show the characteristics measured from the fiber coupled MLL at 25 °C. The black contours show the required coupling losses for uncorrected error free (BER < 1e-12) operation, while the deep blue contours show the requirements for an uncorrected BER of 5e-5 compatible with IEEE standard 802.3bj.

Figure 5 shows 6 scenarios at 14 Gbps. (a) Shows the maximum laser RIN and minimum required line power for different assumptions in regards to *IL*_*A*_ assuming the SOA to be operated in the linear regime at room temperature (25 °C). This serves as a baseline link performance. The RIN and line power measurements done on the MLL^9^ are overlaid on the graph, wherein the RIN corresponds to ${\sigma }_{P}^{2}/{P}_{AV}^{2}$, with ${P}_{AV}$ the optical power and ${\sigma }_{P}$ its standard deviation (std. dev.), obtained by integrating the Power Spectral Density (PSD) between 5 MHz and 20 GHz (since the RIN of the utilized MLL rolls off to shot noise levels above 4 GHz, this accounts for the total RIN of the comb line). It can be seen that *IL*_*A*_ = 5 dB, a 0 dBm line power and an integrated RIN for isolated comb lines of 4e-3 would support a 1e-12 BER, so that an implementation of the link for small channel counts and at room temperature appears realistic (not surprisingly given the experimental results published in ref.^9^).

Note that the power levels of individual comb lines plotted in Fig. 5 contain the MLL to fiber ILs incurred during the MLL characterization (that are redundant with the MLL to chip coupling losses accounted for by *IL*_*A*_ here). We opted not to normalize them out, since increasing the operating temperature of the laser from 25 °C to 55 °C would already result in a (measured) 1 dB drop of line power; this margin can thus be allocated to an increased laser operating temperature (as the temperature of the laser is increased, the spectrum of the laser also narrows, so that the power of the central comb lines is less sensitive on temperature than the power of the entire comb).

(b) Shows a 12-channel configuration assuming balanced channels, wherein the Tx Filter Extinction is assumed to be 15 dB. A number of channels could be allocated to coding overhead to reduce the bit disparity and approach a truly balanced scenario. This would however be associated to an electrical power consumption overhead, both due to the additional high-speed modulation and to the required data processing. Since the extinction is not impacted by SOA saturation in this case, channel count scalability is mainly limited by reduced SOA gain and the ensuing increase of the relative weight of Rx noise, as well as by the increased NF as the SOA approaches saturation.

(c) On the other hand, shows the results for the worst-case analysis corresponding to 12 synchronized channels assuming a perfect (infinite extinction) Tx filter. The effect of SOA saturation is now clearly visible in the inflection of the contour plots at high comb line power levels, to the extent that at high comb line power levels higher insertion losses actually result in lower BER as they reduce saturation. As a consequence, a comb line RIN of 5e-3 appears to be the absolute upper limit at which the system functions error free without FEC irrespectively of the comb line power. Interestingly, once the finite 15 dB extinction of the Tx filter is factored back in (d), the inflections of the curves occur at notably higher laser powers as a consequence of the stabilization of the SOA gain by the unmodulated lines. The additional power entering the SOA as a consequence of the final extinction of unmodulated lines has two consequences: On the one hand, the data pattern dependent gain is stabilized and XGM reduced. On the other hand, the average gain is also reduced. If the additional power supplied by the unmodulated lines is sufficiently low, i.e., the wideband filter extinction sufficiently high, the former effect can be prevalent so that the net effect is beneficial to the overall link budget.

The graphs (d–f) show how the performance requirements for maintaining an error free link (BER < 1e-12) evolve at increasing temperatures. These also correspond to a realistic 15 dB Tx Filter Extinction as well as 12 correlated channels. Reduced gain, reduced saturation power and increased NF are factored in at increased temperatures. At 55 °C, the requirements on the interface losses become very challenging in the absence of other improvements (discussed below). However, assuming interface losses of *IL*_*A*_ = 4.5 dB in that scenario would still result in a signal Q-factor of 3.9 and an uncorrected BER of 5e-5 compatible with the Reed-Solomon FEC used in 100 G Ethernet standards (as per clause 91 of IEEE Standard 802.3bj, based on 5 dB of coding gain, see for example the 100G-CLR4 specifications), as shown by the blue contours. Interestingly, at 55 °C, at the typical laser RIN and line power of 4e-3 and 0 dBm, and at the required ~3 dB interface losses for uncorrected error free operation, the link is equally limited by RIN, ASE and Rx noise, with noise levels ${\sigma }_{0}+{\sigma }_{1}$ at the input of the TIA of respectively 4.3 μA, 4.1 μA and 4.0 μA (the calculated electrical modulation amplitude at the input of the TIA is 46 μA). At increased temperatures, Rx noise becomes equally important as RIN and ASE due to drastically reduced SOA gain.

**Figure 6:**
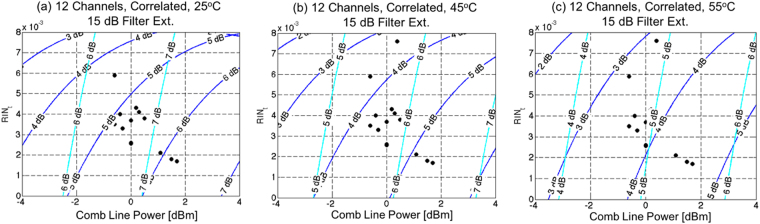
Maximum integrated comb line RIN and required comb line power as a function of the MLL-to-SiP and SOA-to-SiP interface losses (*IL*_*A*_) for a 12-channel link with 25 Gbps serial data rates with the SOA operating at 25 °C, 45 °C and 55 °C assuming correlated channels (worst case) and 15 dB Tx filter extinction. The deep blue contours show the requirements for an uncorrected BER of 5e-5 compatible with IEEE standard 802.3bj and the light blue contours the required characteristics for 3.6e-3 uncorrected BER compatible with 7% overhead hard decision FEC. The dots show the characteristics measured from the fiber coupled MLL at 25 °C.

Not surprisingly, the realization of a 25 Gbps WDM link is significantly more challenging: A link budget analysis shows that the current version of the chip-scale electronics, combined with the SOA and MLL performance, is not sufficient for reliable 25 Gbps multi-channel operation, particularly once the 2.65 dB Rx penalty measured in ref.^9^ at 25 Gbps is taken into account. Under the assumption of improved electronics consisting in a reduction of the Tx rise and fall times to 12 ps and in an elimination of the 2.65 dB Rx penalty, the situation looks much improved, even though some amount of FEC still appears to be required to sustain the link: The deep blue contours in Fig. 6 show the performance requirements for achieving a signal Q-factor of 3.9 and a BER of 5e-5 compatible with the Reed-Solomon FEC used in 100 G Ethernet standards as per clause 91 of IEEE standard 802.3bj for a 12-channel 25 Gbps link (15 dB Tx Filter Extinction, worst case fully correlated data streams) under the assumption of improved electronics (as defined above) and at several SOA temperatures. The light blue contours show the performance metrics required to reach a BER of 3.6e-3 that remains in principle compatible with 7% overhead hard decision FEC^24,25^.

## Discussion

### General Design Guidelines

A couple of general design guidelines can be extracted from this work in regards to the interplay between multi-wavelength comb sources and SOA amplification: As in any SOA amplified WDM link, the SOA gain should be large enough to overcome receiver noise, but not excessive, so as to avoid unnecessary deep saturation of the SOA and resulting high levels of XGM. As a specificity to mode-locked comb sources, the channel spacing is on an exact grid, so that FWM cannot be suppressed by detuning individual channels. Most comb sources come with a number of central, high power lines, as well as lower power, higher noise lines on the sides of the spectrum that are not suitable for error free data transmission. These lower power lines can be both a problem and an opportunity: If the cumulative power contained in them is excessive, a large portion of the available SOA output power (saturation output power) will be allocated to them, leading at best to an inefficient link in terms of overall power consumption and at worst to the link to fail due to insufficient power per channel/insufficient OMA at the receiver input. Thus, some form of wideband filtering will be generally required to select the utilized lines. Complete filtering of these low power lines is however not necessarily the optimum: As shown above, some residual power in these unmodulated lines contributes to stabilizing the SOA gain and to reducing pattern dependencies and XGM. When modulated with complementary data streams intended to stabilize the total SOA input power, these lines can contribute even more effectively to a reduction of XGM. Finally, it should be noted that due to the channel-to-channel correlation of RIN, at least in the case of the investigated MLLs, the comb line RIN cannot be suppressed by means of joint amplification of all the lines by an SOA: As the RIN of a group of several lines can be orders of magnitude smaller than that of isolated comb lines, there will be no correlation between gain fluctuation (XGM) and the intensity noise carried by the carriers. Compared to existing PSM short distance Silicon Photonics links, in which noise is typically dominated by receiver noise and thus maximization of the OMA is the only criterion guiding the biasing of the modulators, in an amplified link both OMA and extinction are important. The high level of RIN of the comb lines further makes extinction an important metric. For these reasons, a tradeoff between OMA and extinction has to be struck in the choice of modulator bias point.

### Outlook and Further Work

A number of measures can be implemented to further improve the optical power budget of the link. Most straightforwardly, one may increase the drive voltage above 2 V_pp_ or combine a dual output driver with a ring assisted MZI to increase the OMA, incidentally also reducing the chirp and thus extending the reach of the link^26^. Implementing MLLs with integrated Distributed Bragg Reflectors (DBRs) with a reduced reflection band might also allow increasing the power per line at the cost of a reduced channel count^27^. Finally, unused comb lines might be used to balance the power entering the SOA.

A module level integration of the transceiver architecture described in this paper will also have to address the sensitivity of MLLs to back-reflections. Micro-packaging solutions compatible with isolator integration have been developed for the hybrid integration of a laser with SiP chips^28^. Heterogeneous integration of the MLL^29^ and the SOA^30^ would also reduce reflections at the first interfaces, potentially allowing for implementation of the system with a single isolator at the output of the Tx. Hybrid integration is even more challenging for the SOA due to its two optical interfaces and is currently under development.

## Conclusions

We have modeled a dense WDM system architecture relying, in addition to integrated SiP devices, on a single semiconductor MLL as a light source as well as a single SOA for reamplification. Single-channel system characterization of an SiP Tx/Rx pair comprising essential devices – an RRM with performance tradeoffs specially targeted for this system architecture, Ge WPDs and commercial FC-PDs, as well as driver and receiver electronics – served to calibrate a link budget further adapted to model the WDM solution. We have introduced channel cross-talk, RIN, SOA based nonlinear effects, and penalties related to handling of polarization diversity into a link budget model for the full WDM system calibrated and verified with the aforementioned single-channel experiments. Assumed device characteristics were based on measured components (0 dBm comb line power, −120 dBc/Hz comb line RIN between 0 and 4 GHz, 16.5 dBm SOA saturation power at 55 °C, optimized SiP RRMs operated with a 2 V_pp_ drive voltage).

We conclude that 12-channel operation at 14 Gbps serial data rates is realistic in cooled operation pending MLL-to-SiP-chip and SOA-to-SiP-chip interface losses better than 3 dB (no channel pre-emphasis, equalization or FEC). Uncorrected, semi-cooled operation with SOA temperatures up to 55 °C appears more challenging, however interface losses of ~4.5 dB remain acceptable if standard Reed-Solomon FEC is implanted as per IEEE standard 802.3bj. With these same insertion losses and assuming a modest improvement of the currently utilized Tx and Rx electronics, 12-channel 25 Gbps operation is expected to be compatible with standard 7% hard decision FEC in semi-cooled operation.

## Electronic supplementary material

Calibrated Link Budget of a Silicon Photonics WDM Transceiver with SOA and Semiconductor Mode-Locked Laser

## References

[1] Liang D, Roelkens G, Baets R, Bowers JE (2010). Hybrid Integrated Platforms for Silicon Photonics. materials.

[2] Lamponi M (2011). Low-threshold heterogeneously integrated InP/SOI lasers with a double adiabatic taper coupler. Photon. Technol. Lett..

[3] Rosales R (2011). InAs/InP quantum-dot passively mode-locked lasers for 1.55 μm applications. J. Sel. Top. Quant. Electron..

[4] Müller, J. *et al*. Silicon photonics WDM transmitter with single section semiconductor mode-locked laser. *Adv. Opt. Technol*. (De Gruyter) **4**(2*)*, 119−145 (2015).

[5] Zheng X (2012). Ultralow power 80 Gb/s arrayed CMOS silicon photonic transceivers for WDM optical links. J. Lightwave Technol..

[6] Pantouvaki, M. *et al*. 8 × 14 Gb/s Si Ring WDM Modulator Array with integrated tungsten heaters and Ge Monitor Photodetectors. In *Proc. 2014 Opt. Fib. Comm. Conf. (OFC)*, Th1C.5.

[7] Gill DM (2015). Demonstration of a High Extinction Ration Monolithic CMOS Integrated Nanophotonic Transmitter and 16Gb/s Optical Link. J. Sel. Top. Quant. Electron..

[8] Zeiler M (2016). A system-level model for high-speed, radiation-hard optical links in HEP experiments based on silicon Mach-Zehnder Modulators. J. Instrument..

[9] Martír, A. *et al*. Silicon Photonics Transmitter with SOA and Semiconductor Mode-Locked Laser. *in review*. 10.1038/s41598-017-14347-3 PMC5654767 29066785

[10] Zhang, L. *et al.* Silicon-Based Microring Resonator Modulators for Intensity Modulation. *J. Select. Top. Quant. Electron*. **6**(1), 149–158 (2010).

[11] Priem G (2005). Optical bistability and pulsating behavior in Silicon-on-Insulator ring resonator structures. Opt. Express.

[12] Diez S (1997). Four-Wave Mixing in Semiconductor Optical Amplifiers for Frequency Conversion and Fast Optical Switching. J. Select. Top. Quant. Electron..

[13] Gutiérez-Castrejón R, Schares L, Duelk M (2008). SOA nonlinearities in 4x25 Gb/s WDM pre-amplified system for 100-Gb/s Ethernet. Opt. Quant. Electron..

[14] Bonk R (2012). Linear semiconductor optical amplifiers for amplification of advanced modulation formats. Opt. Express.

[15] Gay, M. *et al*. Single Quantum Dash Mode-Locked Laser as a Comb-Generator in Four-Channel 112 Gbit/s WDM Transmission. In *Proc. 2014 Opt. Fib. Comm. Conf. (OFC*), -Tu2H.5.F.

[16] Manganelli, C. L., Pintus, P., Gambini, F., Di Pasquale, F. & Oton., C. J. Thermal tuning double ring resonator filters: experimental analysis. In *Proc. 2016 Conf. Group IV Photon*., 12–13.

[17] Laere, V. *et al.* Focusing polarization diversity gratings for Silicon-on-Insulator integrated circuits. In *Proc.**2008 Int. Conf. Group IV Photon.**(GFP)*, 203–205.

[18] Baudot, C. *et al*. Low Cost 300mm Double-SOI Substrate for Low Insertion Loss 1D & 2D Grating Couplers. In *Proc. 2014 Int. Conf. Group IV Photon. (GFP)*, 137–138.

[19] Carroll L, Gerace D, Cristiani I, Andreani LC (2014). Optimizing polarization-diversity couplers for Si-photonics: reaching the -1dB coupling efficiency threshold. Opt. Express.

[20] Sfar Zaoui W, Kunze A, Vogel W, Berroth M (2013). CMOS-Compatible Polarization Splitting Grating Couplers With a Backside Metal Mirror. Photon. Technol. Lett..

[21] Kucharski, D. *et al*. 10 Gb/s 15 mW Optical Receiver with Integrated Germanium Photodetector and Hybrid Inductor Peaking in 0.13μm SOI CMOS Technology. in *Proc. 2010 Int. Sol.-State Circ. Conf. (ISSC*), 360–361.

[22] Moscoso-Mártir, A. *et al*. Hybrid Silicon Photonics Flip-Chip Laser Integration with Vertical Self-Alignment. *Proc. 2017 Conf. Las. Electro-Opt*. *Pacific-Rim* (CLEO-PR) *and 2017 Opto-Electron. Comm. Conf*. (OECC).

[23] Mikkelsen B (1989). Temperature Dependent Gain and Noise of 1.5 μm Laser Amplifiers. Electron. Lett..

[24] Mizuochi T (2006). Recent Progress in Forward Error Correction and Its Interplay With Transmission Impairments. J. Sel. Top. Quant. Electron..

[25] Chang F, Onohara K, Mizuochi T (2010). Forward Error Correction for 100 G Transport Networks. Comm. Mag..

[26] Cardenas J (2013). Linearized silicon modulator based on a ring assisted Mach-Zehnder interferometer. Opt. Express.

[27] Joshi S (2014). Quantum dash based single section mode locked lasers for photonic integrated circuits. Opt. Express.

[28] Snyder B, Corbett B, O’Brien P (2013). Hybrid Integration of the Wavelength-Tunable Laser with a Silicon Photonic Integrated Circuit. J. Lightwave Technol..

[29] Keyvaninia S (2015). Narrow-linewidth short pulse III-V-on-silicon mode-locked lasers based on a linear and ring cavity geometry. Opt. Express.

[30] Wu Y (2016). All-optical NRZ wavelength conversion based on a single hybrid III-V/Si SOA and optical filtering. Opt. Express.

