# Supplementary material for: Calibrated Link Budget of a Silicon Photonics WDM Transceiver with SOA and Semiconductor Mode-Locked Laser
Source: Sci Rep. 2017 Sep 20;7:12004. doi: 10.1038/s41598-017-12023-0 (PMC5607229; doi:10.1038/s41598-017-12023-0)
Supplement: Supplementary file 1 — Calibrated Link Budget of a Silicon Photonics WDM Transceiver with SOA and Semiconductor Mode-Locked Laser [file 41598_2017_12023_MOESM1_ESM.pdf]

Calibrated Link Budget of a Silicon Photonics WDM Transceiver with SOA and Semiconductor Mode-Locked Laser

Supplementary Materials

Alvaro Moscoso-Mártir, Juliana Müller, Elmira Islamova, Florian Merget, and Jeremy Witzens*

Institute of Integrated Photonics (IPH) of RWTH Aachen University, Sommerfeldstr. 24, D-52074 Aachen, Germany. E. Islamova is now with Innolume GmbH.
*jwitzens@iph.rwth-aachen.de

These supplementary materials contain two detailed aspects of the link budget calculations not directly described in the main text of the paper: In section I, we describe the assumptions made for the evaluation of the Four Wave Mixing (FWM) penalty. In section II, we describe the evaluation of optical cross-talk due to spectral overlap of adjacent channels with the Optical Add-Drop Multiplexer (OADM) passband as well as the optimization of the OADM passband.

I. Evaluation of FWM Penalty

Figure SM1 shows the Semiconductor Optical Amplifier (SOA) characterization data on which the evaluation of the FWM penalty is based. Two lines carrying each -15 dBm of power are fed into the packaged SOA (corresponding to ~ -18 dBm after coupling to the SOA chip). This power level corresponds to the maximum expected 1-level power entering the SOA for a given channel, as discussed in section III of the paper. Figure SM1(a) shows the generated power in one of the two spectrally nearest generated lines as a function of the input channel spacing. The expected drop in FWM conversion efficiency is seen as the input channel spacing is successively increased from 100 to 200 and 300 GHz (the relevant spacings for the FWM induced cross-talk for the central channels in a 12-channel configuration). Importantly, the FWM efficiency also drops as a spectrally broad background, generated by the Amplified Spontaneous Emission (ASE) of an Erbium Doped Fiber Amplifier (EDFA) devoid of input signal and emulating the gain saturation induced by the other channels not directly involved in a given FWM process, is added to the input of the SOA. In a 12-channel configuration, this background would be larger than -8 dBm at the input of the packaged SOA, accounting for the other 10 channels assuming an average power per channel of -18 dBm (-21 dBm after coupling to the SOA chip). This power will be even higher in practice due to the partial extinction of the channel selection filter interposed between the Mode-Locked Laser (MLL) and the Resonant Ring Modulators (RRMs), as also discussed in section III of the paper. This filter lets some of the power of the nominally unused comb lines pass through, so that we estimate a background power of -5 dBm to be typical (-8 dBm after coupling to the SOA chip), i.e., just slightly above $P_{sat,in}$.

A worst-case analysis consists in assuming the amplitudes of all the involved FWM processes aggressing a given channel to add up constructively with each other and further to interfere with the aggressed channel in such a way as to both minimize the 1-level amplitude and to maximize the 0-level amplitude. These latter two assumptions are not necessarily contradictory, since the RRMs also introduce phase shifts during modulation and since other elements of the system may drift over time, but clearly constitute a pessimistic worst-case scenario. Deriving the beat note between the aggressed channel and the light generated from other channels by FWM, the penalty is given by

$$-10log_{10}\left(1-2\left[2\sqrt{P_{1,2}}+2\sqrt{P_{2,4}}+\sqrt{P_{3,6}}\right]\cdot\frac{1+\sqrt{\frac{P_{SOA,0}}{P_{SOA,1}}}}{1-\frac{P_{SOA,0}}{P_{SOA,1}}}\right) \quad \text{(SM1)}$$

where $P_{1,2}$ is the relative power generated by two lines spaced by respectively 100 and 200 GHz from the aggressed channel, $P_{2,4}$ is the relative power generated by two lines spaced by respectively 200 and 400 GHz and $P_{3,6}$ is the relative power generated by two lines spaced by respectively 300 and 600 GHz. The amplitudes $\sqrt{P_{1,2}}$ and $\sqrt{P_{2,4}}$ are doubled to account for the fact that a pair of lines at both lower and at higher frequencies than the aggressed optical channel can contribute. $\sqrt{P_{3,6}}$ is only added once, since for a central line (worst case) of a 12-channel configuration there will only be a single line spaced by 600 GHz from the optical carrier. The factor 2 before the square bracket accounts for the beating between the carrier and the FWM contributions. The last term accounts for the finite extinction of the signal at the output of the SOA (the numerator sums the reduction of the 1-level with the increase of the 0-level;

the denominator normalizes the penalty to the initial Optical Modulation Amplitude (OMA)). Even in this worst-case analysis (also taking into account a reduction of the signal extinction from 17 to 14 dB due to SOA saturation in a 12-channel configuration), the FWM penalty is estimated to remain below 0.4 dB. Overall penalties related to FWM remain low as the power contained in any given pair of comb lines is low. The high overall power entering the SOA further contributes to reducing FWM.

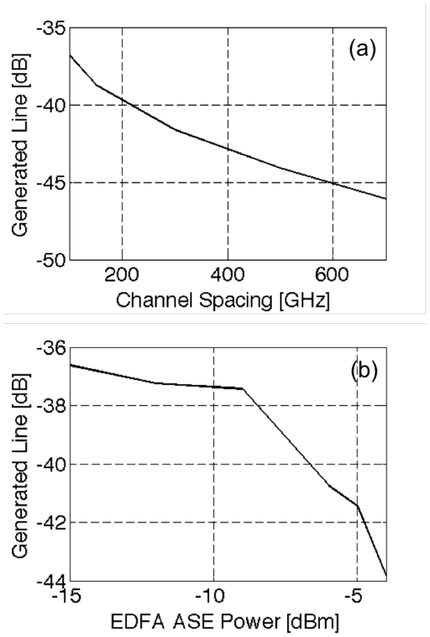

Fig. SM1. Characterization of SOA FWM. (a) Power generated in each of the two nearest generated lines as a function of the frequency spacing of the two injected unmodulated lines, each carrying -15 dBm prior to entering the packaged SOA. In (b) a wide spectrum background is added to the SOA input (ASE generated by an EDFA coupled into the signal path). The frequency spacing between the input lines is maintained at 100 GHz. The power generated in each of the nearest generated lines is plotted as a function of the background power at the input of the SOA. In both cases, the power of the generated lines is indicated relative to the power of the initially injected lines, compared to each other at the output of the SOA. FWM efficiency is seen to drop both with channel spacing and with background power. A sharp decrease in FWM efficiency is visible once a substantial background power pushes the SOA into saturation.

II. OPTIMIZATION OF OADM BANDWIDTH AND EVALUATION OF CROSS-TALK PENALTIES DUE TO SPECTRAL OVERLAP

In order to assess the inter-channel cross-talk penalty associated to the OADM bandwidth, a trapezoid shaped 25 Gbps PRBS-7 signal with the correct rise and fall times is first preconditioned by filtering it 500 times with a low pass filter with a 400 GHz cutoff frequency, yielding a smooth signal shape. It is then filtered by a single pole filter emulating the RRM. Next, the adjacent signals are generated by time shifting the PRBS signal by random amounts and up and down conversion of the optical carrier frequency by 100 GHz. The sum of the three signals is sent through the OADM and Rx filters after which an eye diagram is generated and its vertical opening extracted.

The OADM transfer function is modeled by a Lorentzian as

$$\frac{E_{Drop}}{E_{In}} = \frac{1}{1+i\frac{f-f_r}{\Delta f_{OF}}} \qquad \text{(SM2)}$$

where E_{In} is the amplitude of the light entering the OADM, E_{Drop} is the amplitude of the light coupled to the drop port, f is the frequency of the light, f_r is the resonant frequency of the ring assumed to be equal to the carrier frequency f_0 of the targeted channel, and Δf_{OF} is the equivalent single-sided passband of the OADM (note that the Noise Equivalent Bandwidth (NEB) of the standalone OADM is $\pi/2$ larger). Δf_{OF} is half the Full Width at Half Maximum (FWHM) of the resonator and is thus given by $\Delta f_{OF} = f_0/2Q$, where Q is the loaded Q-factor of the cavity. Note that the Insertion Losses (IL) of the OADM are accounted for separately in the link budget and are thus not considered in Eq. (SM2).

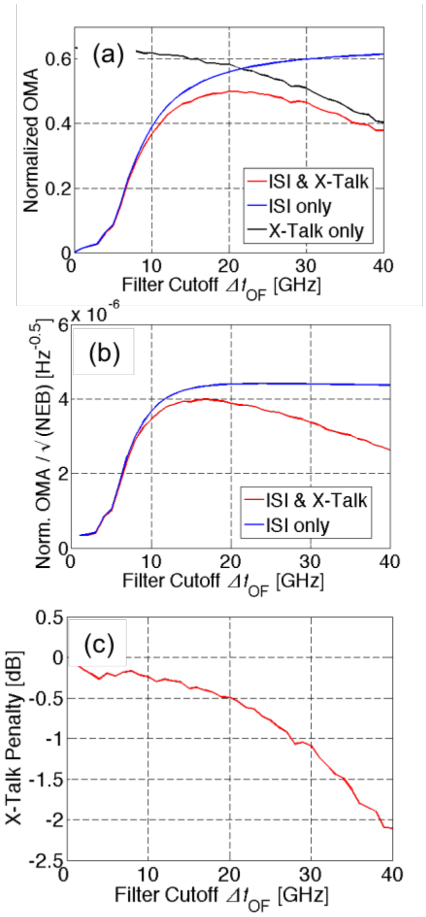

Fig. SM2. Modeling of the effect of the OADM bandwidth on the channel performance. (a) shows the vertical eye height after filtering by the Tx, OADM and Rx (blue curve). The red curve also incorporates the effect of inter-channel cross-talk from adjacent channels due to the finite OADM bandwidth. The black curve corresponds to results when the targeted channel is only filtered by the Tx and Rx (i.e., ignoring OADM induced ISI) and is representative for the cross-talk penalty. In (b) the ISI only and full model curves from (a) are divided by the square root of the NEB (as applied to signal-ASE beat noise) resulting from the OADM and Rx filters. This number is characteristic for the signal quality if ASE-signal beat noise is the dominating noise term. (c) shows the cross-talk penalty at a fixed OADM bandwidth and corresponds to the ratio of the red and blue curves from (a). This curve is directly used as a vertical eye opening penalty in the full link model. In all three graphs the x-axis corresponds to the equivalent single sided cutoff frequency of the OADM (Δf_{OF}).

Figure SM2(a) shows the results. The computed vertical eye opening (normalized OMA, given as a fraction of the input signal) taking into account Intersymbol Interference (ISI) arising from the 16 ps driver rise and fall times, an 18.5 GHz RRM bandwidth (approximated as single pole), the varied OADM bandwidth (single pole) and the 21 GHz Rx bandwidth (third order Butterworth), as well as taking into account channel cross-talk due to spectral overlap, is shown in red. Note that SOA Cross-Grain Modulation (XGM), FWM, various ILs and the modulation penalty are taken into account separately in the link budget, as this analysis only serves to determine the optimum OADM bandwidth in view of ISI and spectral channel overlap. For comparison, the vertical eye opening obtained by considering ISI only, i.e., without adding the adjacent channels, is shown in blue. The black curve shows the effect of cross-talk without considering OADM induced ISI, computed by adding the adjacent channels to the targeted channel, but applying the OADM transfer function only to the adjacent channels and not to the targeted channel. This way, the targeted channel ISI suffers only from the Transmitter (Tx) Electro-Optic (E/O) and Receiver (Rx) Opto-Electronic (O/E) filters, but not from the OADM bandwidth. The red curve combines the effect of cross-talk and OADM induced ISI and is thus limited by the blue curve (OADM ISI) for low Δf_{OF} and by the black curve (cross-talk) for large Δf_{OF}.

The best vertical eye opening is obtained at $\Delta f_{OF} = 20$ GHz (i.e., for a 40 GHz optical filter bandwidth) and is 1 dB below the highest vertical eye opening obtained with an infinite bandwidth OADM in the absence of adjacent channels. The system level penalty compared to single-channel operation is however not quite as high once all factors are considered. Indeed, reducing the OADM bandwidth also contributes to reducing ASE noise. Figure SM2(b) shows the vertical eye opening for ISI only and for ISI + cross-talk penalty, both divided by the square root of the NEB resulting from the OADM and the Rx filter functions. This factor would be proportional to the signal Q-factor if the

noise were uniquely due to ASE-signal beat noise. The penalty between the best-case single-channel operation and the operation with cross-talk to adjacent channels and $\Delta f_{OF} = 20$ GHz then reduces to 0.4 dB. Since in practice the two dominant noise sources in this link are ASE and Relative Intensity Noise (RIN), the latter not significantly impacted by the OADM transfer function as it decays to shot noise levels above 4 GHz, the actual effective system penalty is in between these two values.

Figure SM2(c) shows the cross-talk penalty as applied to the system at a specific OADM single sided bandwidth Δf_{OF} (and simply corresponds to the ratio of the red and blue curves in Fig. SM2(a)). This function can be directly imported in the full link model and applied as a penalty to the final eye height arriving at the Rx (note that this is a bounded penalty and not a noise term). It reaches 0.5 dB at $\Delta f_{OF} = 20$ GHz. In this model, the ISI penalty right after the Tx is calculated as 1 dB at 25 Gbps, consistent with the measurements reported in [6]. After the OADM it further increases to 1.7 dB, to which the 2.65 dB data rate dependent Rx-electronics penalty measured in [6] is added in the link budget.

A different type of channel cross-talk resides in the weak modulation of an optical carrier by a RRM nominally assigned to an adjacent channel due to a finite overlap of the RRM's Lorentzian transfer function with the optical carrier frequency. This inter-channel cross-talk is calculated as -25 dB between adjacent channels based on the optical carrier detuning, channel spacing and on the Q-factors of the RRMs, and is quite low due to the high attenuation applied by the main on-channel RRM. There is, however, a non-negligible penalty associated to the (average) attenuation of the optical carrier by the RRMs of the two adjacent channels, that cumulatively amounts to 0.5 dB additional ILs. It should be noted that channel cross-talk in cascaded RRM based systems has also been extensively experimentally studied by M. Ashkan Seyedi et al. with results reported in [SM1].

References:

[SM1] M. Ashkan Seyedi, C.-H. Chen, M. Fiorentino, and R. Beausoleil, Error-free DWDM transmission and crosstalk for a silicon photonics transmitter. *Opt. Express* **23**(26), 32968-32976 (2015).
